# Aggregachromic Fluorogenic Asymmetric Cyanine Probes for Sensitive Detection of Heparin and Protamine

**DOI:** 10.3390/molecules30030570

**Published:** 2025-01-27

**Authors:** Anton Kostadinov, Aleksey Vasilev, Stanislav Baluschev, Katharina Landfester

**Affiliations:** 1 Max Planck Institute for Polymer Research, Ackermannweg 10, 55128 Mainz, Germanybalouche@mpip-mainz.mpg.de (S.B.); 2 Faculty of Chemistry and Pharmacy, University of Sofia “Saint Kliment Ohridski”, 1 James Bourchier Blvd., 1164 Sofia, Bulgaria; ohtavv@chem.uni-sofia.bg; 3 Laboratory of Functional and Nanostructured Polymers, Institute of Polymers, Bulgarian Academy of Sciences, Akad. G. Bonchev Str., Bl.103A, 1113 Sofa, Bulgaria; 4 Faculty of Physics, University of Sofia “Saint Kliment Ohridski”, 5 James Bourchier Blvd., 1164 Sofia, Bulgaria

**Keywords:** heparin sensor, protamine sensor, fluorescence probe

## Abstract

The precise and fast detection of heparin, the most widely used anticoagulant, remains a significant challenge for assessing its use in a clinical setting. In this work, we adapt a well-established asymmetric cyanine fluorogenic platform for the purpose of ultrasensitive heparin detection in the presence of common interferant chemical species. Three analogous fluorescence probes are synthesized in order to optimize for the number of binding moieties. Their interaction with heparin is studied using steady-state absorption, fluorescence, and circular dichroism spectroscopy. The obtained probes exhibit a highly sensitive “turn-on” fluorescence response to heparin, with a LOD in the 10–25 nM range, well within practical requirement, as well as a visible colorimetric change. The heparin–probe complex is also employed as a sensitive detection platform for protamine, both in the “turn-off” fluorescence and ratiometric detection schemes.

## 1. Introduction

Heparin is a linear glycosaminoglycan, which consists primarily of trisulfated disaccharide repeating units, and has the highest negative charge density among all known biopolymers. Heparin plays a role in the regulation of several biological processes, including cell growth and regulation, blood clotting, and inflammation [1]. It exhibits high binding affinity towards antithrombin, resulting in enhanced inhibition of the activity of thrombin and other clotting factors. Thus, heparin is employed as an anticoagulant drug in both prophylactic and therapeutic applications [2,3]. For the prevention of thrombosis during surgery and for postoperative and long-term care, the recommended heparin dosages are 2–8 IU/mL (17–67 μM) and 0.2–1.2 IU/mL (1.7–10 μM), respectively. [4] Dosages exceeding the therapeutic window could induce complications such as thrombocytopenia and hemorrhaging [5,6]. In order for safe heparin levels in the blood to be maintained, the fast and reliable detection of heparin concentration is crucial.

Numerous analytical methodologies for the detection and quantification of heparin were developed based on various strategies, including electrochemical methods [7], colorimetric assay [8,9,10,11,12], surface-enhanced Raman spectroscopy [13,14,15], capillary electrophoresis [16], and amperometric [17] and fluorescence methods [18,19,20,21,22,23,24,25,26,27]. Several of the reported fluorescence detection methods rely on a “turn off” signal-response, which may often prove unreliable due to possible quenching by interfering compounds, normally present in the analyzed sample. In order to obtain better selectivity, a “turn on” method of detection would be advantageous [28,29,30].

In this article, we report the synthesis and study of the photophysical properties of newly developed asymmetric monomethine cyanine dyes—molecular sensors for the visible detection of heparin. The new dyes are characterized by negligible intrinsic fluorescence, high binding affinity, and significant fluorescence enhancement in the presence of heparin as well. In the absence of a binding target, both segments of the probe conjugated system exhibit a degree of rotational freedom with respect to each other via their connecting single bond. In this state, when light absorption occurs, excited state electron energy is expended primarily by means of a non-radiative torsional relaxation, thus low fluorescence intensity is observed.

**Figure 1:**
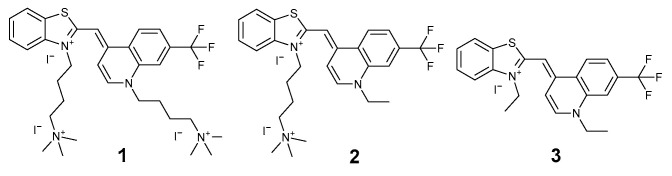
Chemical structures of fluorogenic probes with varying number of cationic binding moieties.

Taking into consideration the exceptionally high negative charge density of heparin, we designed our sensor by functionalizing one or both segments of a thiazole orange (TO) fluorophore with butyl-trimethylammonium moieties (Figure 1). In this way, we hope to induce aggregation on the surface of the heparin molecule. Additionally, by providing one or two cationic functions, we succeed in enhancing the water solubility of the molecules and reducing their self-aggregation in aqueous solutions of the neat dyes. The introduction of a trifluoromethyl group into the chromophore should provide a red shift in the long-wavelength maximum compared to TO, ensuring a visible difference between the free and bound states of the new molecular sensors. For more significant bioaggregation properties, the introduction of a trifluoromethyl group into the chromophore, on the other hand, aims to increase the hydrophobicity of the chromophore part itself.

**Figure 2:**
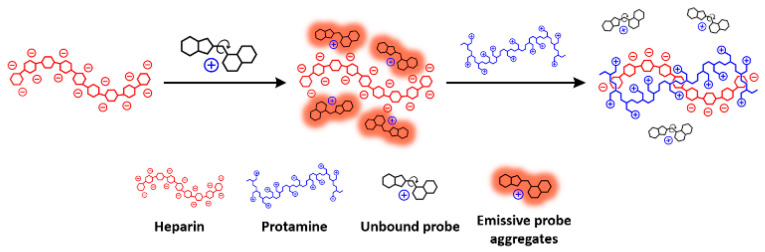
Schematic representation of dye–heparin–protamine binding interaction.

Regarding fluorescence, we expected that, in the presence of heparin, the ammo-nium groups of the molecular sensor bind to the negatively charged sulfate groups of heparin due to Coulombic attraction. Thus, the possibility for intramolecular rotation could be limited, and excited state electrons decay mainly by means of fluorescence emission. As a result, a significant fluorescence enhancement should be observed, allowing for the sensitive detection and quantification of heparin in a model solution (Figure 2).

## 2. Results and Discussion

### 2.1. Photophysical Properties

**Figure 3:**
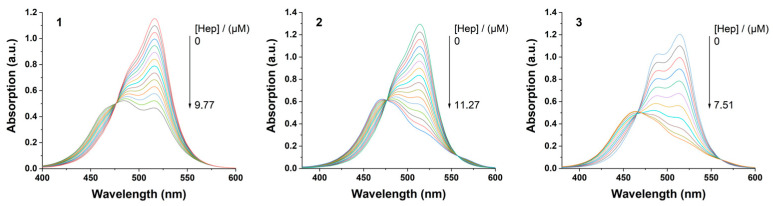
UV-vis spectra of probes **1**–**3** (20 µM), respectively, upon titration with heparin in Tris-HCl buffer (10 mM, pH 7.4).

Changes in the absorption spectra of each probe were observed upon titration with heparin (Figure 3). The observed hypsochromic shift in the absorption maxima of the fluorescent probes, as well as the large Stokes shift in probes **1** and **3** (146 nm), are definitely indicative of the formation of molecular H-aggregates in the presence of heparin. In the proximity of the highly negatively charged heparin backbone, electrostatic repulsion between the anionic probes is partially compensated, allowing the probes to aggregate into dimers or further, into oligomers.

Additionally, in all dyes from the series, a hypochromic shift in the absorption band of unbound probes in solution occurs, corresponding linearly to the concentration of added heparin. For the purpose of molecular recognition based on aggregachromism, chromophores with low self-aggregation in solution are considered most suitable.

**Figure 4:**
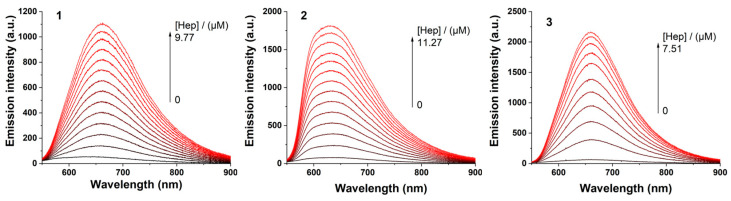
Photoluminescence spectra of probes 1–3 (20 µM), respectively, upon titration with heparin solution in Tris-HCl buffer (10 mM, pH 7.4). Excitation wavelength for probe **1** and **2**: 466 nm; for probe **3**: 461 nm.

As can be seen in Figure 4 and Figure S5, the lowest intensity signal for the presence of initial H-aggregates in TE buffer is characterized by probe **2**, which has two positive charges in the molecule and one butyl-trimethylammonium ion bound to the nitrogen of the benzothiazole. In this probe (**2**), the decrease in the intensity of the chromophore is the most significant, and the characteristic peak for trifluoromethyl analog **3** H-aggregate is not observed. In the case of probe **1** (Figure 3), with increasing heparin concentration, the signal for the monomeric dye remains the least changed in comparison to **2** and **3**, which surprisingly suggests a weaker interaction with heparin. Regarding the UV-VIS absorption analysis of heparin in a model system, the most contrasting picture is with probe **2** (Figure 3), which in our opinion makes it the best visual sensor in the current series.

A well-known characteristic of the TO fluorophore is its intrinsic low fluorescence quantum yield, owing to the possibility for nonemissive torsional relaxation of its singlet excited state. Specifically, when the molecular rotor action of the fluorophore is restricted, a corresponding increase in photoluminescence is observed. This property can be seen translated to the emission spectra of probes **1**–**3** shown in Figure 4. Each probe exhibits very weak intrinsic emission and, upon titration with heparin, a linearly proportional increase in emission intensity is measured within a range of heparin concentrations.

### 2.2. Circular Dichroism Properties of Heparin–Probe Aggregates

**Figure 5:**
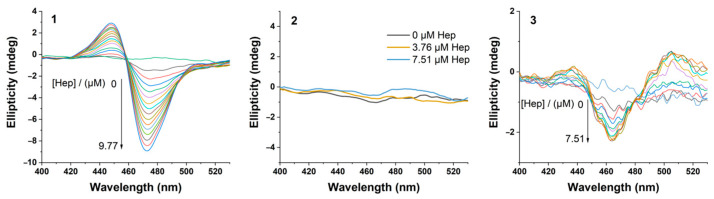
CD spectra of probes **1**–**3** (20 µM) at increasing concentrations of heparin in Tris-HCl buffer (10 mM, pH = 7.4).

A common feature of various chromophore aggregates is the emergence of an electronic circular dichroism (CD) signal, otherwise absent in solutions of free dye molecules. Such signal correlates with the molecules in the aggregate arranging into a chiral formation. In the case of probe **1**, as seen in Figure 5, a strong negative Cotton effect (CE) is recorded at longer wavelengths, as well as a weaker, positive CE that corresponds to the absorption maximum of the probe–heparin complex. Using the exciton chirality rule, the sense of chirality of the aggregate could be inferred [31,32]. A longer wavelength negative CE, followed by a shorter-wavelength positive CE, is indicative of a left-handed helical arrangement of the probe molecules along the heparin backbone. In the case of probe **2**, as seen in Figure 5, no significant electronic CD signal emerges upon the addition of heparin to the probe solution. Taking into consideration the difference in the photoluminescence spectrum of probe **2** (Figure 4), compared to the other two probes, namely the lower Stokes shift, it could be reasoned that the molecules of probe **2** appear to form distinct, nonchiral aggregates in the proximity of heparin.

A weak electronic CD signal Is elicited upon the addition of heparin to a solution of probe **3** (Figure 5). The recorded spectrum represents a multiple CE curve, with a first broad positive CE band centered around 505 nm, followed by a negative CE band, corresponding to the absorption maximum of the probe–heparin complex, as well as a weak positive band, peaking at 438 nm. The observed signals indicate the formation of a right-handed helical aggregate being formed along the heparin backbone.

### 2.3. Heparin Quantification

**Figure 6:**
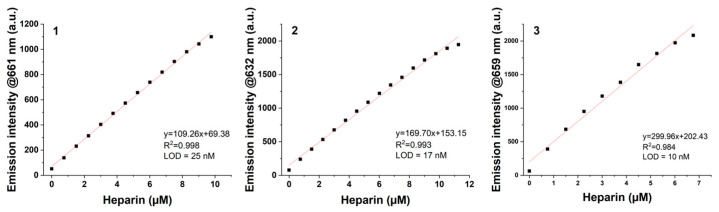
Increase of fluorescence intensity of probes **1**–**3** (20 µM), at their respective emission maxima, with increasing concentration of heparin in Tris-HCl buffer (10 mM, pH = 7.4). Red line represents best fit of calibration curve for signal–response of each probe, obtained by linear regression.

Each of the studied probes exhibits a different range of heparin concentrations, suitable for heparin detection and quantification with the widest range being provided by probe **2**: 0–11.27 µM (Figure 6). The highest degree of fluorescence enhancement at the upper end of their linear signal–response range exhibited probe **3**: 33.0-fold; followed by probe **2**: 23.5-fold; and probe **1**: 21.6-fold. Limit of detection (LOD) was determined using the following formula:

LOD = 3.3 × *σ*/*S*, (1)

where *σ* is the standard deviation obtained from 20 blank measurements; and the factor *S* represents the slope of the calibration curve.

**Figure 7:**
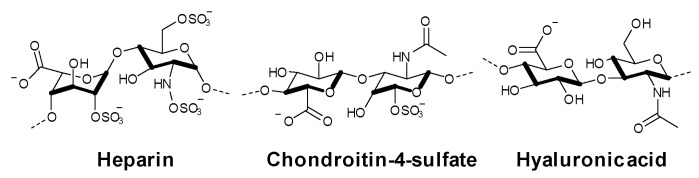
Main disaccharide repeat units of heparin, chondroitin-4-sulfate, and hyaluronic acid.

The selective detection and quantification of heparin in biological samples could potentially be hindered in the presence of structurally similar interferants, namely other sulfated glycosaminoglycans, e.g., chondroitin sulfate and hyaluronic acid. (Figure 7).

**Figure 8:**
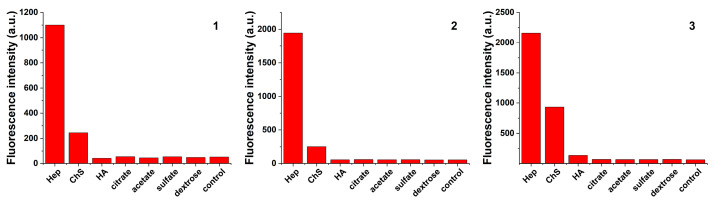
Fluorescence emission intensity of probes **1**–**3** (20 µM) at their respective emission maxima, in Tris-HCl buffer (10 mM, pH = 7.4), in presence of heparin, ChS, HA, sodium citrate, sodium acetate, sodium sulfate, and dextrose at 10 µM concentrations.

To assess the selectivity of probes **1**–**3** in regard to heparin detection, the degree of fluorescence enhancement of each probe was measured when titrated with ChS, HA, and various chemical species commonly present in biological samples (Figure 8).

### 2.4. Protamine Quantification

**Figure 9:**
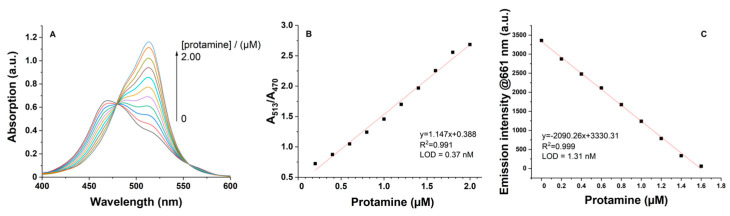
(**A**) UV-Vis spectra of probe **2** (20 µM) heparin complex (1.5 IU/mL) upon titration with protamine in Tris-HCl buffer (10 mM, pH = 7.4); (**B**) ratiometric response in absorption of probe **2** (20 µM) heparin complex (1.5 IU/mL) upon titration with protamine in Tris-HCl buffer (10 mM, pH = 7.4); (**C**) change in fluorescence intensity of probe **2** upon addition of increasing concentrations of protamine in Tris-HCl buffer (10 mM, pH = 7.4).

Heparin overdose is associated with many adverse and potentially life-threatening side effects. In order to reduce the activity of heparin in patients, protamine, being the only clinically approved antidote to heparin, is administered. The highly cationic nature of protamine facilitates electrostatic binding to heparin with exceptional affinity, forming a complex and thereby inactivating it. Considering the clinical importance of protamine, its accurate dosing is crucial for neutralizing excess amounts of heparin in patients. Upon introducing protamine to a solution of fluorescence probe and heparin, the absorption spectrum of the probe–heparin complex gradually reverses into that of the unbound probe, indicating competitive displacement of the probe by protamine from the heparin scaffold (Figure 9A). The two distinct absorption maxima of unbound probe in solution and probe–heparin complex allow for ratiometric quantification of added protamine, as shown on Figure 9B. This detection scheme is characterized by a linear signal–response range of 0–2.0 µM and a protamine LOD of 0.37 nM for probe **2**. Moreover, a reduction in the emission Intensity of the probe–heparin complex is observed, linearly corresponding to the amount of added protamine (Figure 9C).

## 3. Experimental Section

### 3.1. Materials and Methods

All chemicals and reagents were obtained from commercial sources and used without further purification unless otherwise stated. 1,4-diiodobutane was purchased from AlfaAesar (Karlsruhe, Germany). 4-chloro-7-trifluoromethylquinoine was purchased from BLDpharm (Shanghai, China). Heparin (199 IU/mg) was purchased from Thermo Fischer Scientific (Fair Lawn, NJ, USA). Protamine sulfate and hyaluronic acid sodium were purchased from Sigma (Taufkirchen, Germany).

^1^H-NMR spectra and ^13^C-NMR spectra were measured on a Bruker Avance 500 MHz spectrometer in DMSO-d_6_ (Bruker, Hanau, Germany). ^19^F-NMR spectra were measured on a Bruker Avance 400 MHz spectrometer in DMSO-d_6_ (Bruker, Hanau, Germany). Melting points were determined using an SMP40 melting point apparatus (Stuart Scientific, Redhill, UK). MALDI-TOF measurements were performed on a Bruker rapifleX MALDI-TOF/TOF mass spectrometer (Bruker, Hanau, Germany). Absorption and fluorescence spectra were obtained on a Duetta Bio Fluorescence and Absorbance Spectrometer (HORIBA, Kyoto, Japan). Circular dichroism spectra were obtained on a JASCO-1500 circular dichroism spectrometer (Jasco, Pfungstadt, Germany).

### 3.2. Synthesis of Compounds

**Scheme 1:**
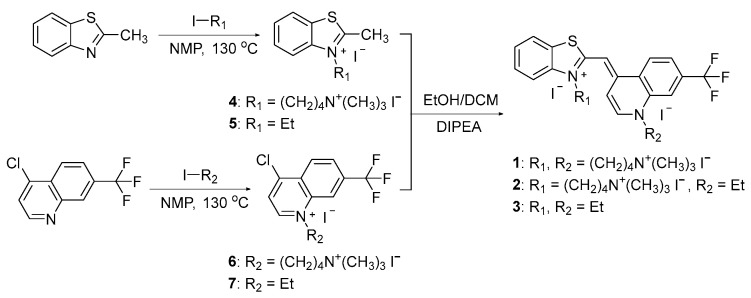
Synthetic route for preparation of fluorogenic heparin probes **1**, **2**, and **3**.

Probes **1**–**3** and their synthetic intermediates have been prepared by following the procedure represented in Scheme 1.

2-((7-(trifluoromethyl)-1-(4-(trimethylammonium)butyl)quinolin-4(1*H*)-ylidene)methyl)-3-(4-(trimethylammonium)butyl)benzo[d]thiazol-3-ium iodide (**1**):

Intermediate compounds **4** (1.00 g, 1.93 mmol) and **6** (1.16 g, 1.93 mmol) were dissolved in a 10 mL mixture of equal volumes of ethanol and dichloromethane. DIPEA (0.74 mL, 4.25 mmol) was added dropwise. The obtained precipitate was concentrated under reduced pressure. Filtration and washing with cold ethanol yielded the product in the form of a red powder (510 mg, 28.0%). Melting point > 250 °C. ^1^H-NMR (500 MHz, DMSO-d_6_), δ (TMS, ppm): 9.02–9.00 (d, 1H), 8.65–8.64 (d, 1H), 8.33 (s, 1H), 8.10–8.09 (d, 1H), 7.95–7.93 (d, 1H), 7.89–7.87 (d, 1H), 7.64–7.61 (t, 1H), 7.47–7.44 (t, 1H), 7.41–7.39 (d, 1H), 6.94 (s, 1H), 4.71–4.67 (t, 4H), 3.38–3.31 (m, 4H), 3.02 (s, 18 H), 1.98–1.92 (m, 2H), 1.79–1.73 (m, 6 H). ^13^C-NMR (125 MHz, ppm), δ (TMS, ppm): 161.32, 148.33, 145.68, 140.23, 137.49, 133.07, 132.75, 129.07, 128.55, 127.00, 125.77, 124.81, 123.71, 122.56, 115.91, 114.01, 109.22, 89.35, 65.23, 65.10, 53.83, 52.85 ((-N^+^(CH_3_)_3_)_2_), 45.99, 26.17, 24.49, 20.03, 19.98. ^19^F-NMR (376 MHz, DMSO-d_6_), δ (TMS, ppm): 61.11. MS (MALDI-TOF): calculated for C_32_H_44_F_3_N_4_S, *m*/*z*: 573.3239; found, 573.2336.

2-((1-ethyl-7-(trifluoromethyl)quinolin-4(1*H*)-ylidene)methyl)-3-(4-(trimethylammonium)butyl)benzo[d]thiazol-3-ium iodide (**2**):

Intermediate compounds **4** (669 mg, 1.29 mmol) and **7** (500 mg, 1.29 mmol) were dissolved in a 10 mL mixture of equal volumes of ethanol and dichloromethane. DIPEA (491 µL, 2.84 mmol) was added dropwise. The obtained precipitate was concentrated under reduced pressure. Filtration and washing with cold ethanol yielded the product in the form of a red powder (419 mg, 43.6%). Melting point > 250 °C. ^1^H-NMR (500 MHz, DMSO-d_6_), δ (TMS, ppm): 9.05–9.03 (d, 1H), 8.71–8.70 (d, 1H), 8.42 (s, 1H), 8.16–8.14 (d, 1H), 8.01–7.99 (d, 1H), 7.93–7.91 (d, 1H), 7.71–7.67 (t, 1H), 7.53–7.50 (t, 1H), 7.48–7.47 (d, 1H), 6.99 (s, 1H), 4.76–4.71 (m, 4 H), 3.43–3.40 (t, 2H), 3.08 (s, 9H), 1.99–1.96 (m, 2H), 1.85–1.79 (m, 2H), 1.49–1.46 (t, 3H). ^13^C-NMR (125 MHz, ppm), δ (TMS, ppm): 161.14, 148.36, 145.40, 140.26, 137.31, 133.09, 132.76, 128.99, 128.43, 127.01, 125.65, 124.77, 123.70, 122.49, 116.02, 113.88, 109.47, 89.12, 65.25, 52.86 (-N^+^(CH_3_)_3_), 49.96, 45.89, 24.47, 20.00, 15.13. ^19^F-NMR (376 MHz, DMSO-d_6_), δ (TMS, ppm): 61.22. MS (MALDI-TOF): calculated for C_27_H_32_F_3_N_3_S, *m*/*z*: 487.3084; found, 487.2269.

3-ethyl-2-((1-ethyl-7-(trifluoromethyl)quinolin-4(1*H*)-ylidene)methyl)benzo[d]thiazol-3-ium iodide (**3**)

Intermediate compounds **5** (398 mg, 1.30 mmol) and 7 (503 mg, 1.30 mmol) were dissolved in a 10 mL mixture of equal volumes of ethanol and dichloromethane. DIPEA (494 µL, 2.86 mmol) was added dropwise. The obtained precipitate was concentrated under reduced pressure. Filtration and washing with cold ethanol yielded the product in the form of a red powder (354 mg, 51.3%). Melting point > 250 °C. ^1^H-NMR (500 MHz, DMSO-d_6_), δ (TMS, ppm): 9.04–9.02 (d, 1H), 8.67–8.65 (d, 1H), 8.38 (s, 1H), 8.14–8.12 (d, 1H), 7.99–7.97 (d, 1H), 7.88–7.87 (d, 1H), 7.69–7.66 (t, 1H), 7.51–7.48 (t, 1H), 7.43–7.41 (d, 1H), 7.01 (s, 1H), 4.77–4.69 (m, 4H), 1.48–1.45 (t, 3H), 1.42–1.39 (t, 3H). ^13^C-NMR (125 MHz, ppm), δ (TMS, ppm): 160.79, 148.20, 145.16, 139.91, 137.26, 133.03, 132.71, 129.05, 128.30, 126.98, 125.59, 124.90, 123.63, 122.43, 115.83, 113.76, 109.24, 88.96, 49.86, 41.98, 15.06, 12.94. ^19^F-NMR (376 MHz, DMSO-d_6_), δ (TMS, ppm): 61.27. MS (MALDI-TOF): calculated for C_22_H_20_F_3_N_2_S, *m*/*z*: 401.1299; found, 401.2010.

### 3.3. Heparin Sensing

Stock solutions of heparin, chondroitin sulfate (ChS), and hyaluronic acid (HA) were prepared with a concentration of 750 µM in Tris-HCl buffer (10 mM, pH = 7.4). The protamine stock solution used had a concentration of 100 µM, prepared in the same buffer. Titration was performed by adding stock solutions of the respective biopolymer in 3 µL increments, into a 10 mm quartz cuvette, containing 3 mL, 20 µM dye solution with continuous gentle stirring. Calculations of the molarity of heparin and ChS solutions were performed using the mass of their main disaccharide repeat unit: 665 g/mol for heparin and 500 g/mol for ChS.

### 3.4. Protamine Sensing

A stock solution of protamine with a concentration of 100 µM was prepared in Tris-HCl buffer (10 mM, pH = 7.4). Titration was performed by adding the stock solution in 6 µL increments, to a 10 mm quartz cuvette, containing 3 mL 20 µM dye solution and 7.56 µM (1 IU/mL) heparin.

## 4. Conclusions

In this work, three novel fluorescence probes were designed, synthesized, and employed for the ultrasensitive detection and quantification of heparin and protamine within a clinically relevant range of concentrations. All three fluorescent probes exhibited heparin LOD in the nanomolar range. An inverse correlation between the number of cationic binding moieties and heparin LOD was observed. In addition, electronic circular dichroism measurements revealed a divergence in the chiral sense of the aggregates that the studied probes form in proximity of the heparin backbone. Among the probes in this work, probe **2** exhibited a superior linear detection range for heparin, as well as insensitivity towards a variety of interfering chemical species, commonly present in clinical samples. The probe offers several desirable sensing modalities, rivaling and even surpassing in their characteristics those of several other sensing platforms currently present in the published literature.

## Data Availability

Data are contained within the article and Supplementary Materials.

## Supplementary Materials

The following supporting information can be downloaded at: https://www.mdpi.com/article/10.3390/molecules30030570/s1. Table S1: Spectral characteristics of probes **1**–**3** in presence of heparin. Figures S1–S3: Excitation spectra of probes **1**–**3** (20 µM) before and after addition of 1 IU/mL Hep in Tris-HCl buffer (10 mM, pH = 7.4). Figure S4: Emission intensity of probes 1–3 at increasing concentration, in presence of 2 IU/mL Hep in Tris-HCl buffer (10 mM, pH = 7.4). Figure S5: Absorption spectra of 10 µM TE buffer solution of probe 1 neat, and in presence of dsDNA, RNA, or heparin. Figures S6–S8: ^1^H-NMR spectra of compounds **1**–**3** in DMSO-d_6_.; Figures S9–S11: ^13^C-NMR spectra of compounds **1**–**3** in DMSO-d_6_. Figures S12–S14: ^19^F-NMR spectra of compounds 1–3 in DMSO-d6. Figures S15–S17: MALDI-TOF spectra of probes **1**–**3**.
