# Supplementary material for: Aggregachromic Fluorogenic Asymmetric Cyanine Probes for Sensitive Detection of Heparin and Protamine
Source: Molecules. 2025 Jan 27;30(3):570. doi: 10.3390/molecules30030570 (PMC11820481; doi:10.3390/molecules30030570)
Supplement: Supplementary file 1 [file molecules-30-00570-s001.zip › molecules-3410419-supplementary.pdf]

Supplementary information

Aggregachromic fluorogenic asymmetric cyanine probes for sensitive detection of heparin and protamine

Anton Kostadinov [1], Aleksey Vasilev [2], Stanislav Baluschev [1,3] and Katharina Landfester [1,*]

[1] Max Planck Institute for Polymer Research, Ackermannweg 10, Mainz 55128, Germany; kostadinova@mpip-mainz.mpg.de
[2] Faculty of Chemistry and Pharmacy, University of Sofia "Saint Kliment Ohridski", 1 James Bourchier blvd., Sofia 1164, Bulgaria; ohtavv@chem.uni-sofia.bg
[3] Faculty of Physics, University of Sofia "Saint Kliment Ohridski", 5 James Bourchier blvd., Sofia 1164, Bulgaria; balouche@mpip-mainz.mpg.de
[4] Laboratory of functional and nanostructured polymers, Institute of Polymers, Bulgarian Academy of Sciences, Akad. G. Bonchev Str., Bl.103A, 1113 Sofa, Bulgaria.
* Correspondence: landfest@mpip-mainz.mpg.de;

Content

Synthesis of intermediates

Table S1 – Spectral characteristics of probes 1-3

Figures S1-S3 - Excitation spectra of probes **1-3** (20 µM) before and after the addition of 1 IU/mL Hep in Tris-HCl buffer (10 mM, pH = 7.4).

Figure S4 Emission intensity of probes **1-3** at increasing concentration, in the presence of 2 IU/mL Hep in Tris-HCl buffer (10 mM, pH = 7.4).

Figure S5 - Absorption spectra of 10 µM TE buffer solution of probe **1** neat, and in the presence of dsDNA, RNA, or heparin.

Figures S6-S8 - ^{1}H-NMR spectra of compounds **1-3** in DMSO-*d*$_6$.

Figures S9-S11 - ^{13}C-NMR spectra of compounds **1-3** in DMSO-*d*$_6$.

Figures S12-S14 – ^{19}F-NMR spectra of compounds **1-3** in DMSO-*d*$_6$.

Figures S15-S17 - MALDI-TOF spectra of probes **1-3**.

Synthesis of intermediates

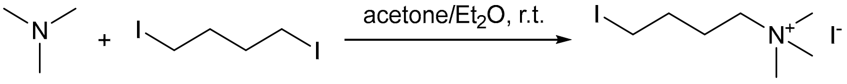

4-iodo-N,N,N-trimethylbutyl-1-ammonium iodide. Trimethylamine (1.37 g, 23.2 mmol) and 1,4-diiodobutane (28.75 g, 92.8 mmol) were dissolved in 50 mL mixture of equal volumes acetone and diethyl ether. Continuous stirring at room temperature for 24 h led to the formation of a white precipitate, which upon filtration, washing with diethyl ether and drying, yielded the product in the form of white crystals (8.36 g, 97.7%).

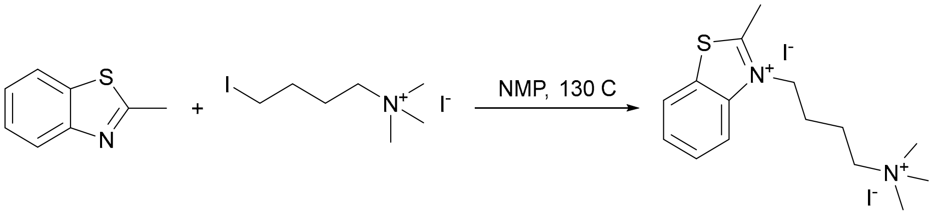

2-methyl-3-(4-(trimethylammonium)butyl)benzo[d]thiazol-3-ium iodide (4). 2-methylbenzo[d]thiazole (0.857 g, 5.74 mmol) and 4-iodo-N,N,N-trimethylbutyl-1-ammonium iodide (2.12 g, 0.00574 mol) and 1 mL N-methylpyrrolidone (NMP) were added to a sealed pressure tube. The reaction mixture was stirred continuously at 130 °C for 3 h. After cooling down to ambient temperature, the reaction product was then filtered off and washed with 20 mL diethyl ether, yielding **4** in the form of a green powder (2.59 g, 87.9 %).

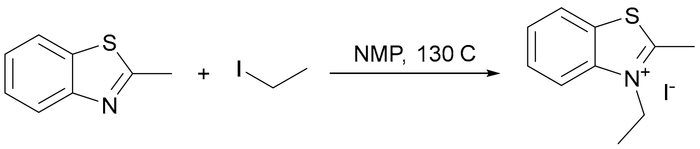

3-ethyl-2-methylbenzo[d]thiazol-3-ium iodide (5). 2-methylbenzo[d]thiazole (2.00 g, 13.4 mmol) and ethyl iodide (6.27 g, 40.2 mmol) were added to a sealed pressure tube. The reaction mixture was stirred continuously at 130 °C for 3 h. After cooling down to ambient temperature, the reaction product was then filtered off and washed with 20 mL diethyl ether, yielding **5** in the form of a pale purple powder (1.24 g, 30.2 %).

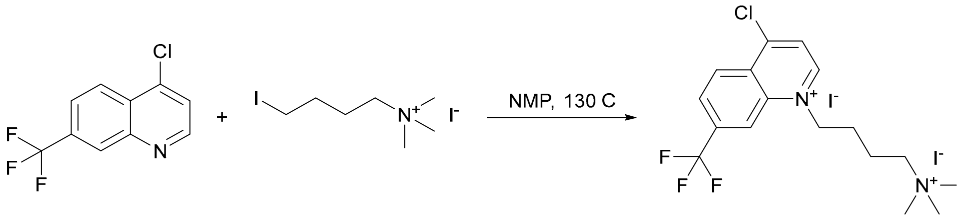

4-chloro-7-(trifluoromethyl)-1-(4-(trimethylammonium)butyl)quinolin-1-ium iodide (6). 4-chloro-7-(trifluoromethyl)quinoline (1.00 g, 4.32 mmol) and 4-iodo-N,N,N-trimethylbutyl-1-ammonium iodide (1.59 g, 4.32 mmol) were added to 1 mL NMP in a sealed pressure tube. The reaction mixture was stirred continuously at 130 °C for 3 h, then cooled down to ambient temperature, washed with 20 mL diethyl ether and dried, yielding **6** in the form of a yellow powder (1.36 g, 52.5 %).

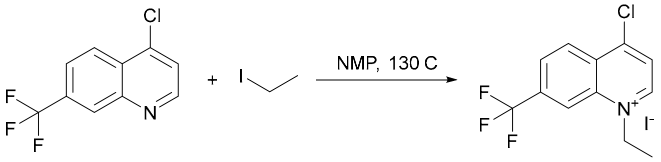

4-chloro-1-ethyl-7-(trifluoromethyl)quinolin-1-ium iodide (7). 4-chloro-7-(trifluoromethyl)quinoline (2.36 g, 10.2 mmol) and ethyl iodide (4.76 g, 30.5 mmol) were added to a sealed pressure tube. The reaction mixture was stirred continuously at 130 °C for 3 h, then cooled down to ambient temperature, washed with 30 mL diethyl ether and dried, yielding **7** in the form of a light green powder (2.66 g, 67.4 %).

Table S1 – Spectral characteristics of probes 1-3 in the presence of heparin

probe	$\lambda_{abs.\ max.}$	$\lambda_{em.\ max.}$	Stokes shift	$\Phi_{heparin}$
**1**	516 nm	662 nm	146 nm	0.00566
**2**	514 nm	632 nm	118 nm	0.00508
**3**	514 nm	660 nm	146 nm	0.00755

Excitation spectra of probes 1-3

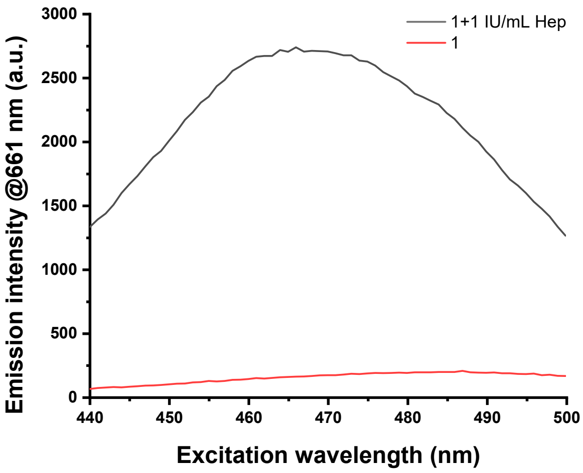

Figure S1 Excitation spectra of probe **1** (20 μM) before and after the addition of 1 IU/mL Hep in Tris-HCl buffer (10 mM, pH = 7.4).

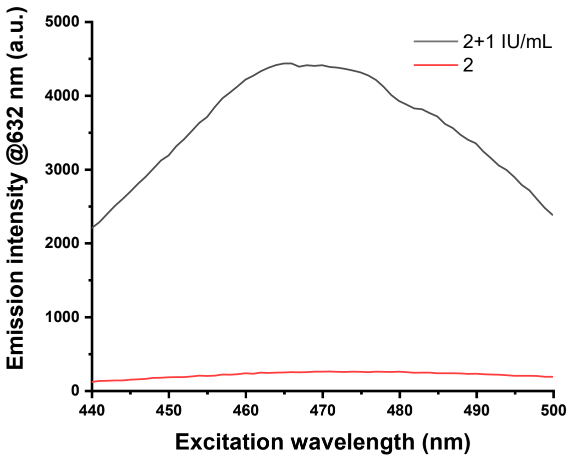

Figure S2 Excitation spectra of probe **2** (20 µM) before and after the addition of 1 IU/mL Hep in Tris-HCl buffer (10 mM, pH = 7.4).

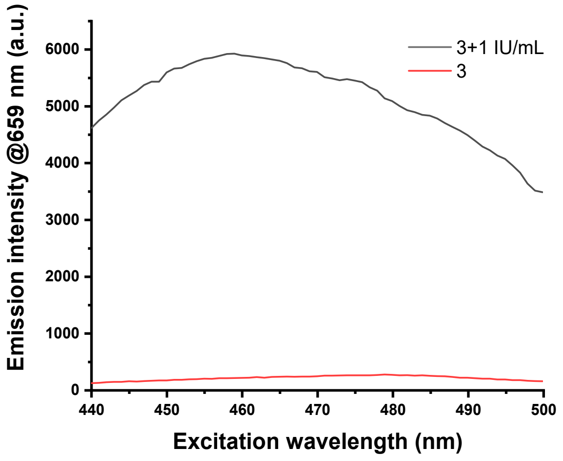

Figure S3 Excitation spectra of probe **3** (20 µM) before and after the addition of 1 IU/mL Hep in Tris-HCl buffer (10 mM, pH = 7.4).

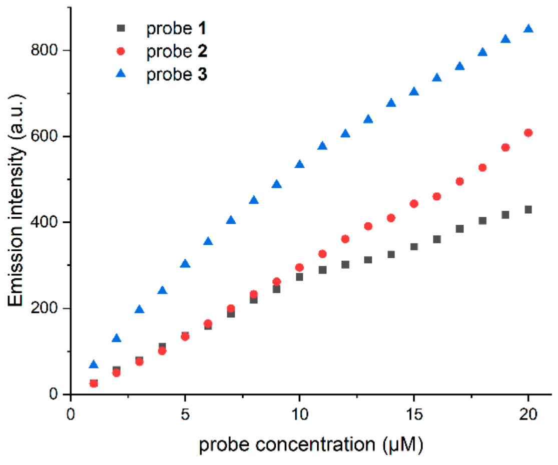

Figure S4 Emission intensity of probes **1-3** at increasing concentration, in the presence of 2 IU/mL Hep in Tris-HCl buffer (10 mM, pH = 7.4).

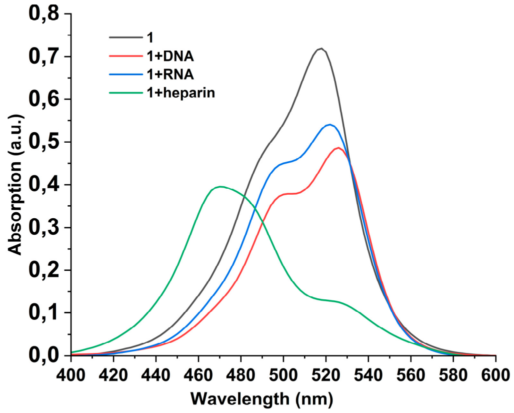

Figure S5 Absorption spectra of 10 µM TE buffer solution of probe **1** neat, and in the presence of dsDNA, RNA, or heparin.

NMR spectra of probes 1-3

^{1}H-NMR spectra have been recorded on Bruker Avance 500 MHz NMR spectrometer. ^{13}C-NMR spectra have been recorded on Bruker Avance 500 MHz NMR spectrometer using attached proton test (APT) procedure. ^{19}F-NMR spectra have been recorded on Bruker Avance 400 MHz NMR spectrometer.

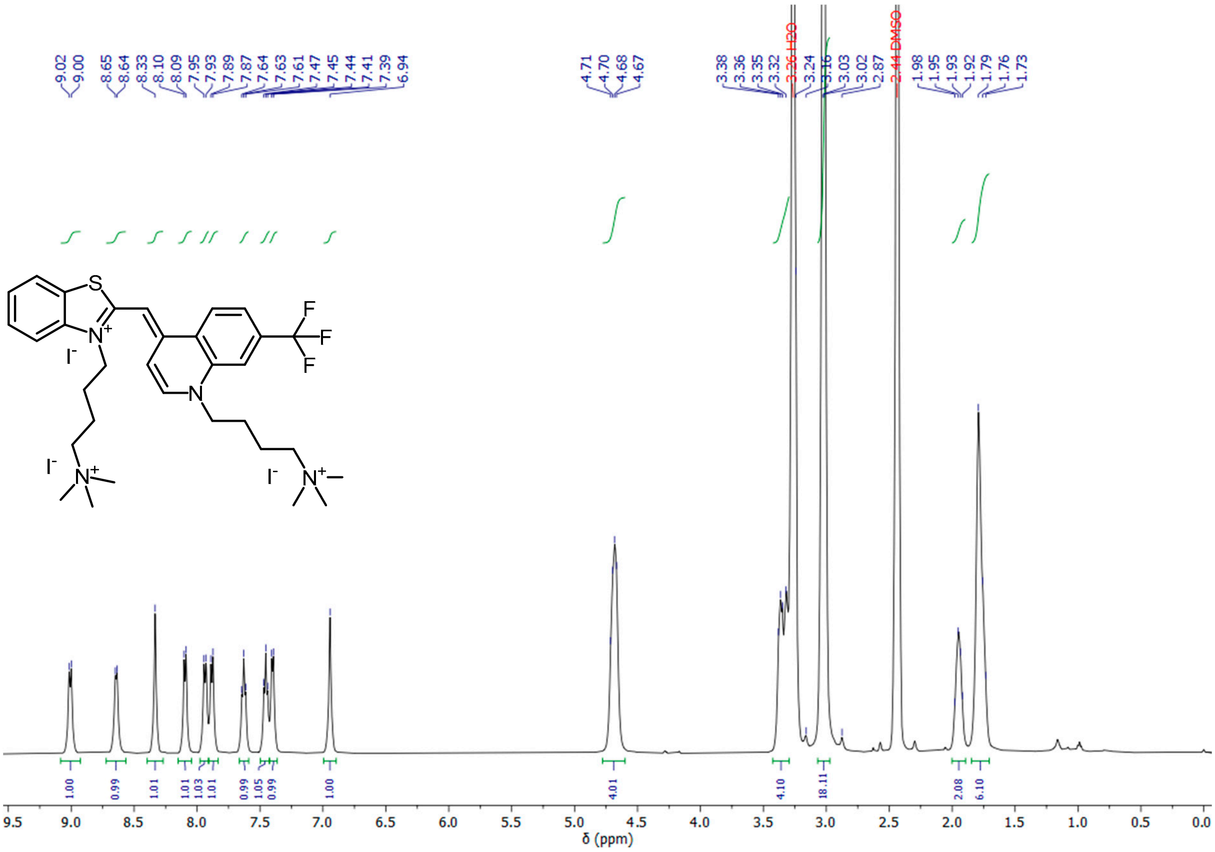

Figure S6 – ^{1}H-NMR spectrum of compound **1** in DMSO-d_6

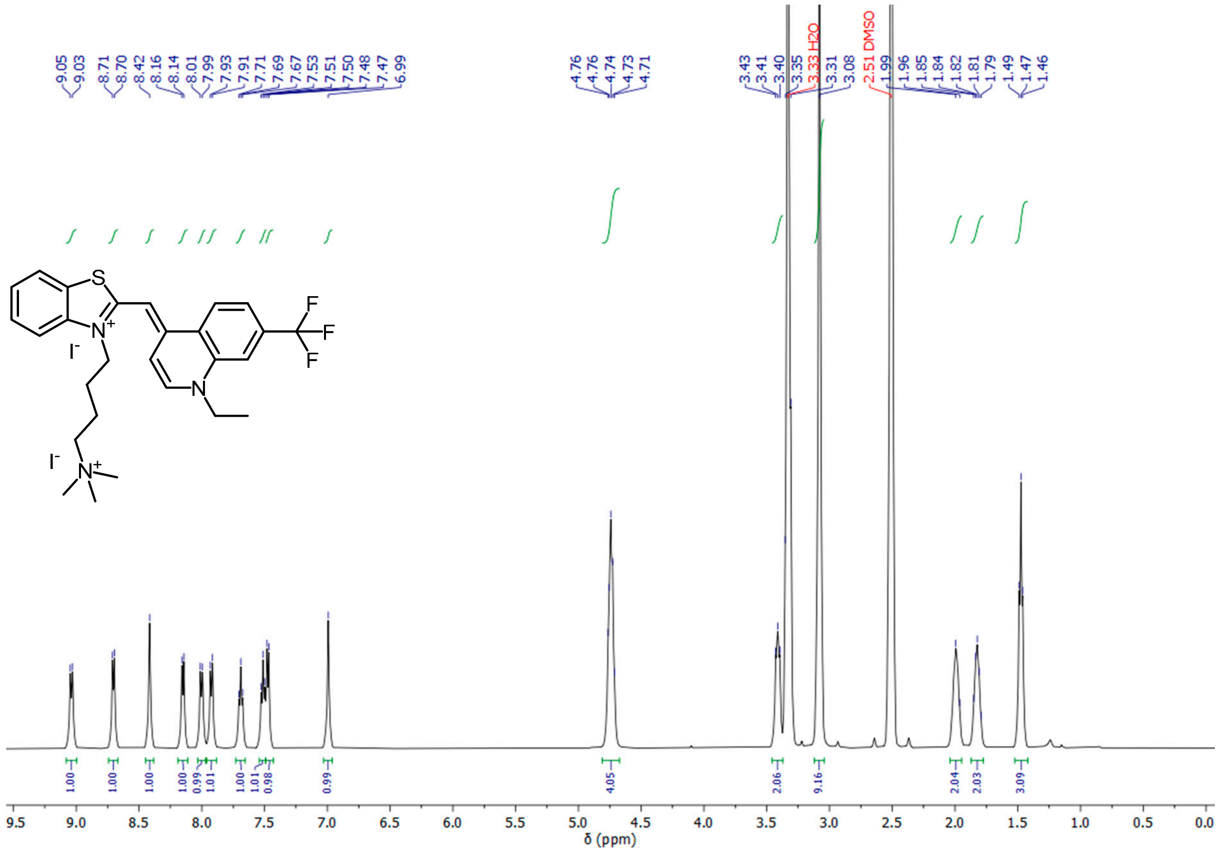

Figure S7 – ^{1}H-NMR spectrum of compound **2** in DMSO-*d*6

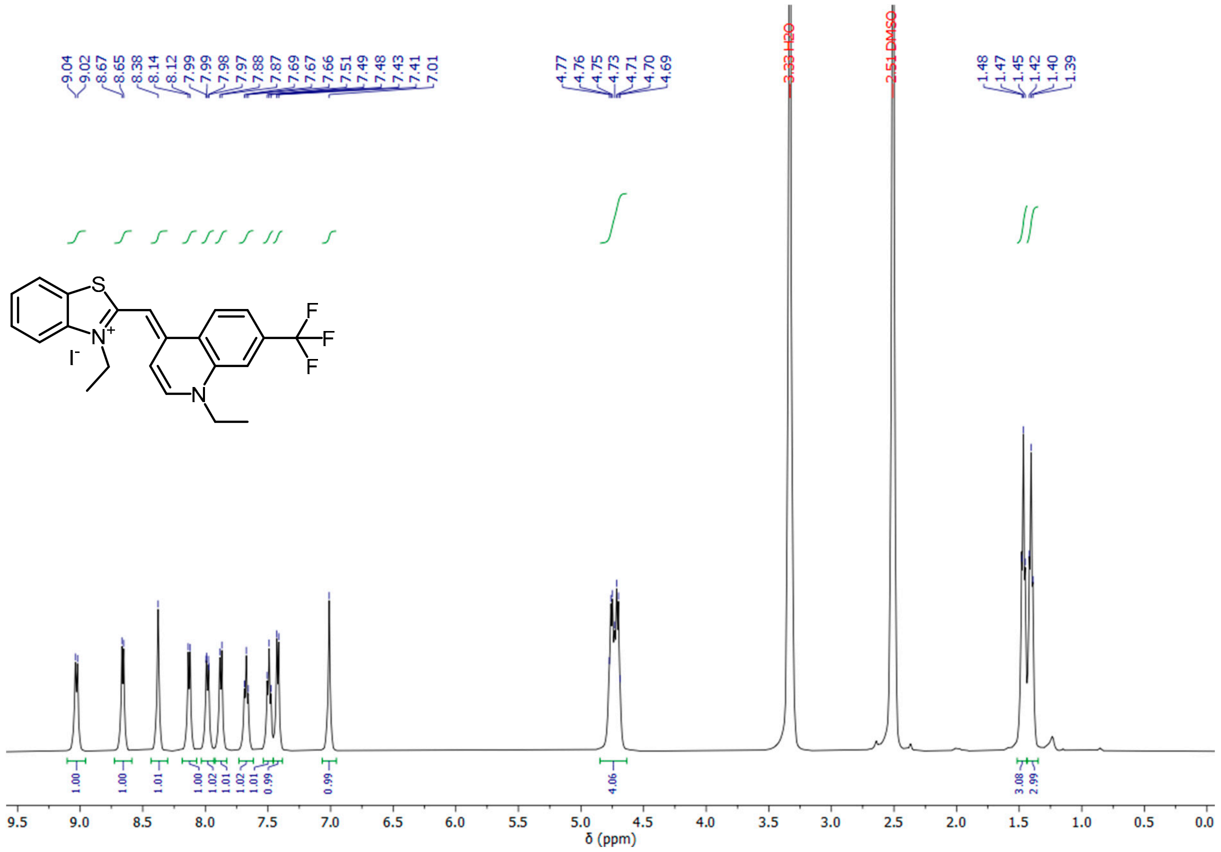

Figure S8 – ^{1}H-NMR spectrum of compound **3** in DMSO-*d*6

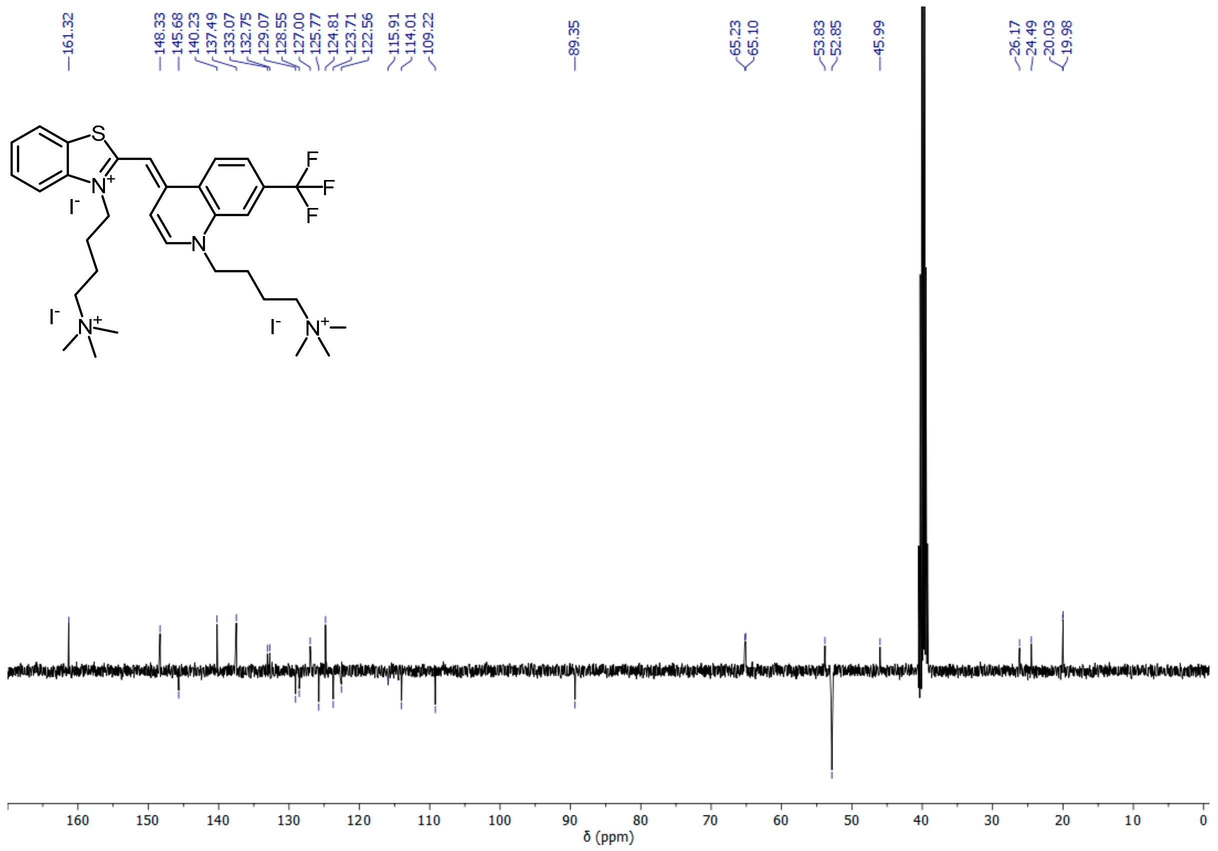

Figure S9 – ^{13}C-NMR spectrum of compound **1** in DMSO-*d6*

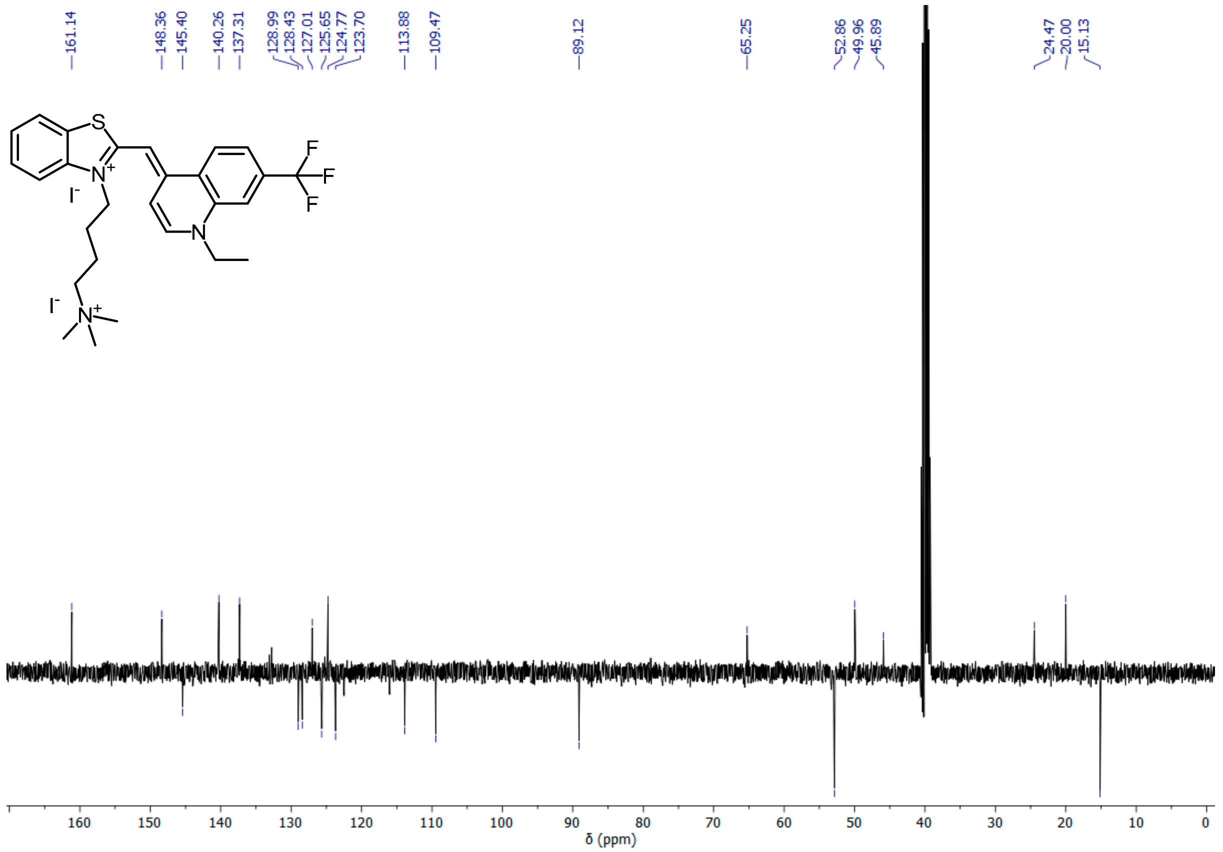

Figure S10 – ^{13}C-NMR spectrum of compound **2** in DMSO-*d6*

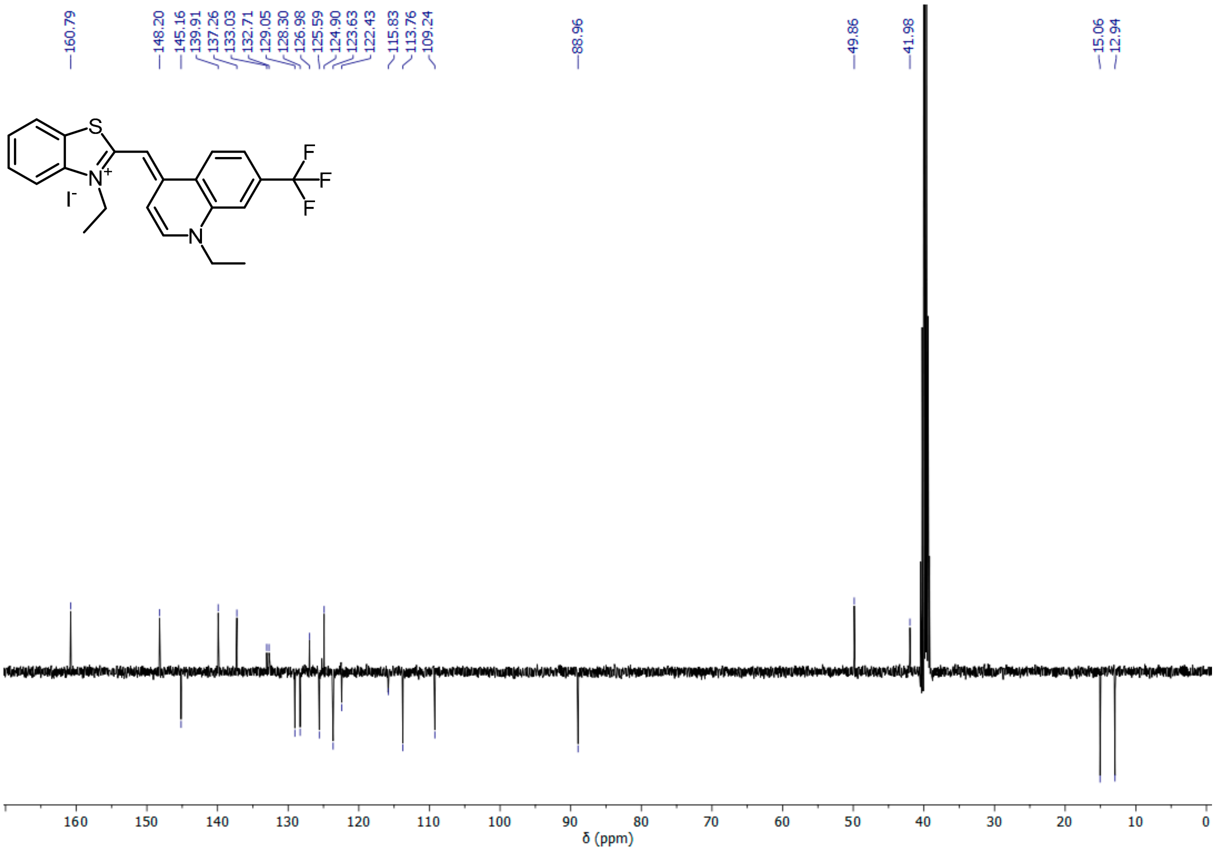

Figure S11 – ^{13}C-NMR spectrum of compound **3** in DMSO-d_6

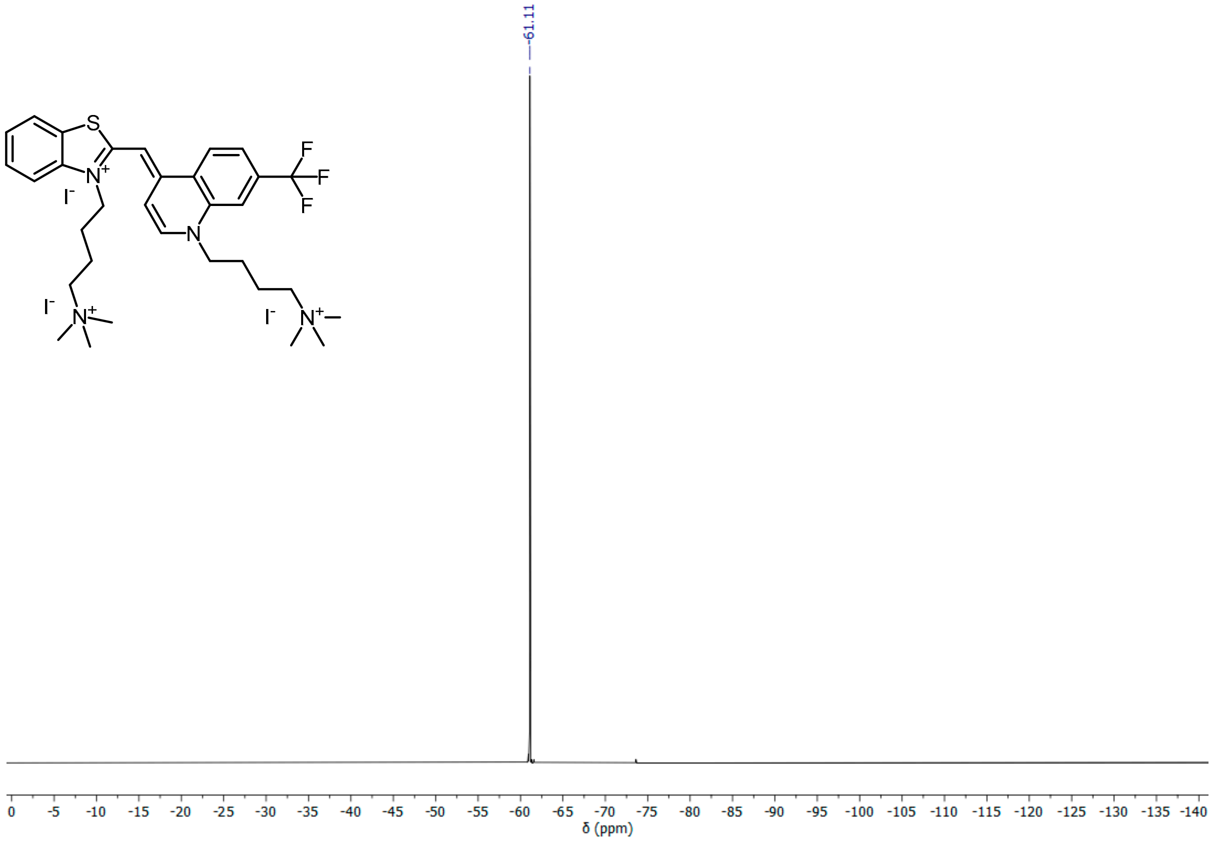

Figure S12 – ^{19}F-NMR spectrum of compound **1** in DMSO-d_6

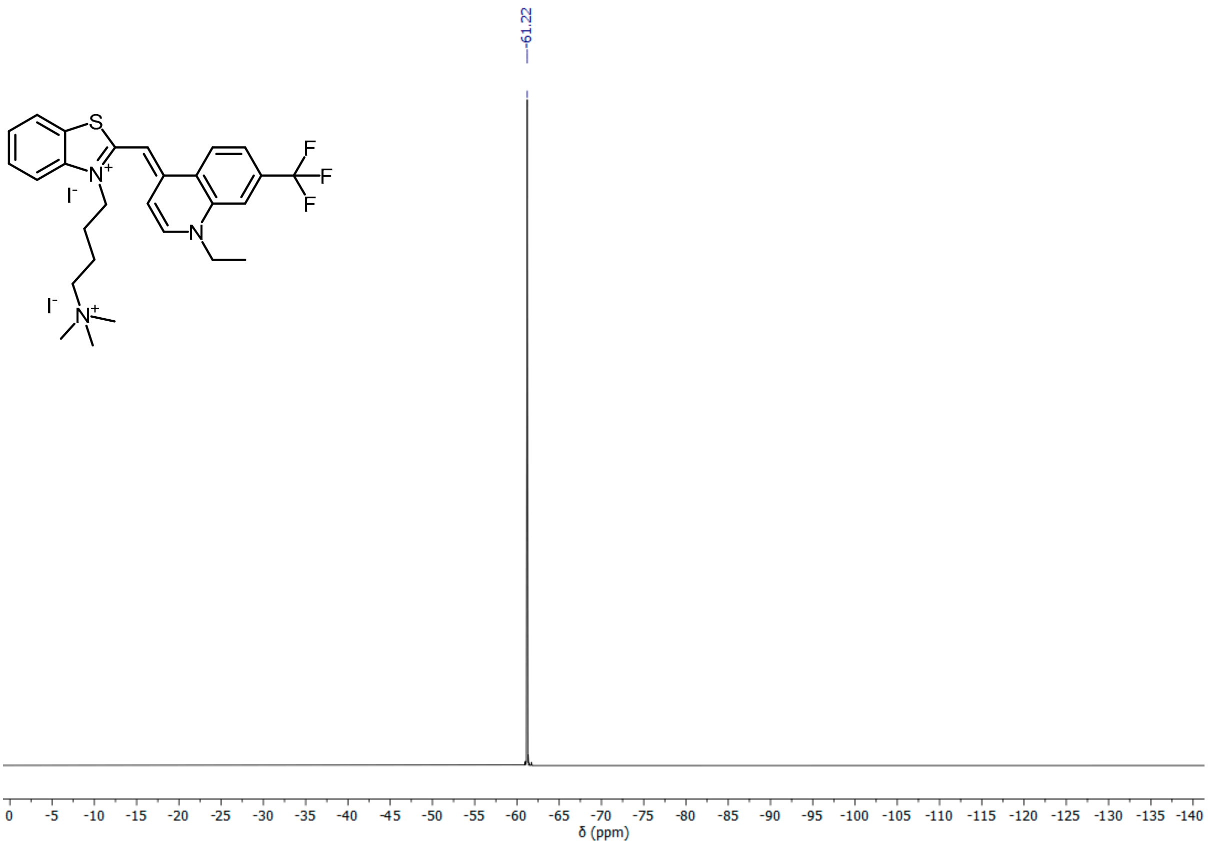

Figure S13 – ^{19}F-NMR spectrum of compound **2** in DMSO-*d6*

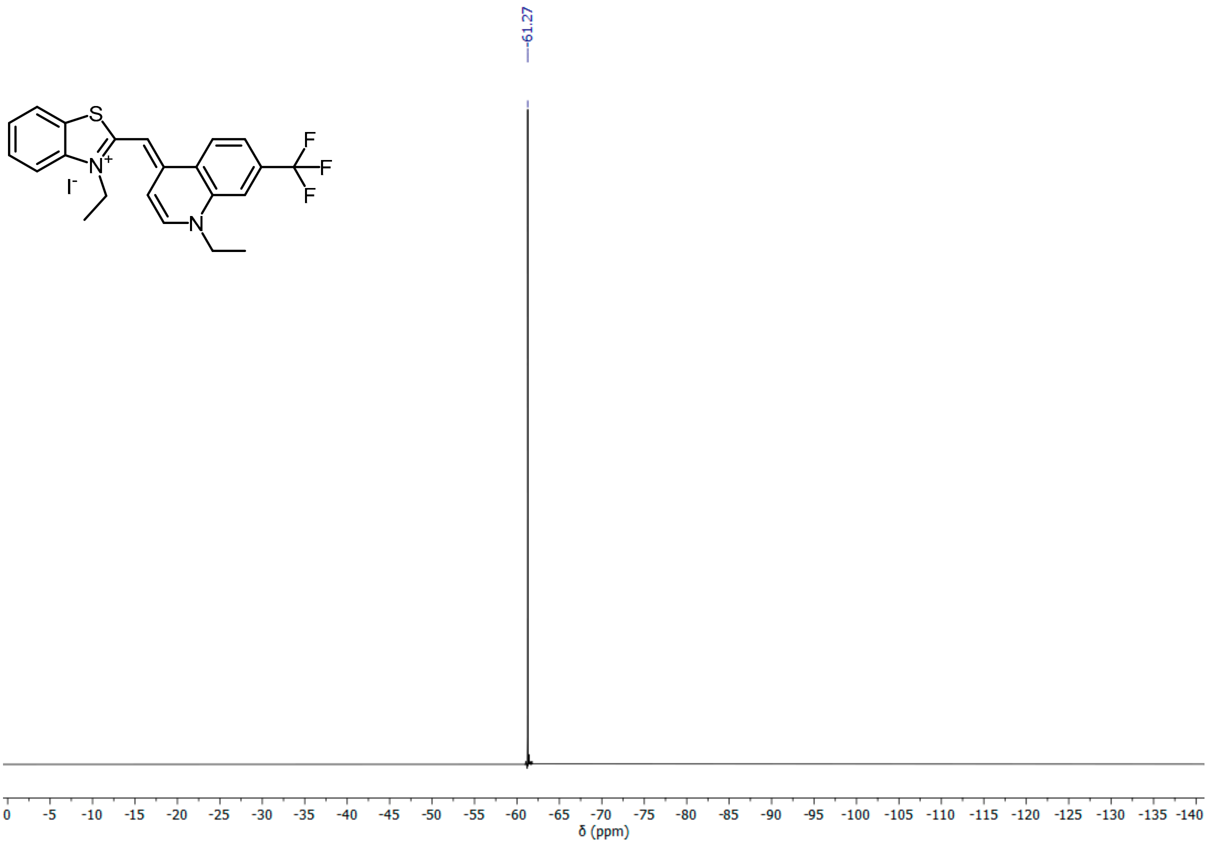

Figure S14 – ^{19}F-NMR spectrum of compound **3** in DMSO-*d6*

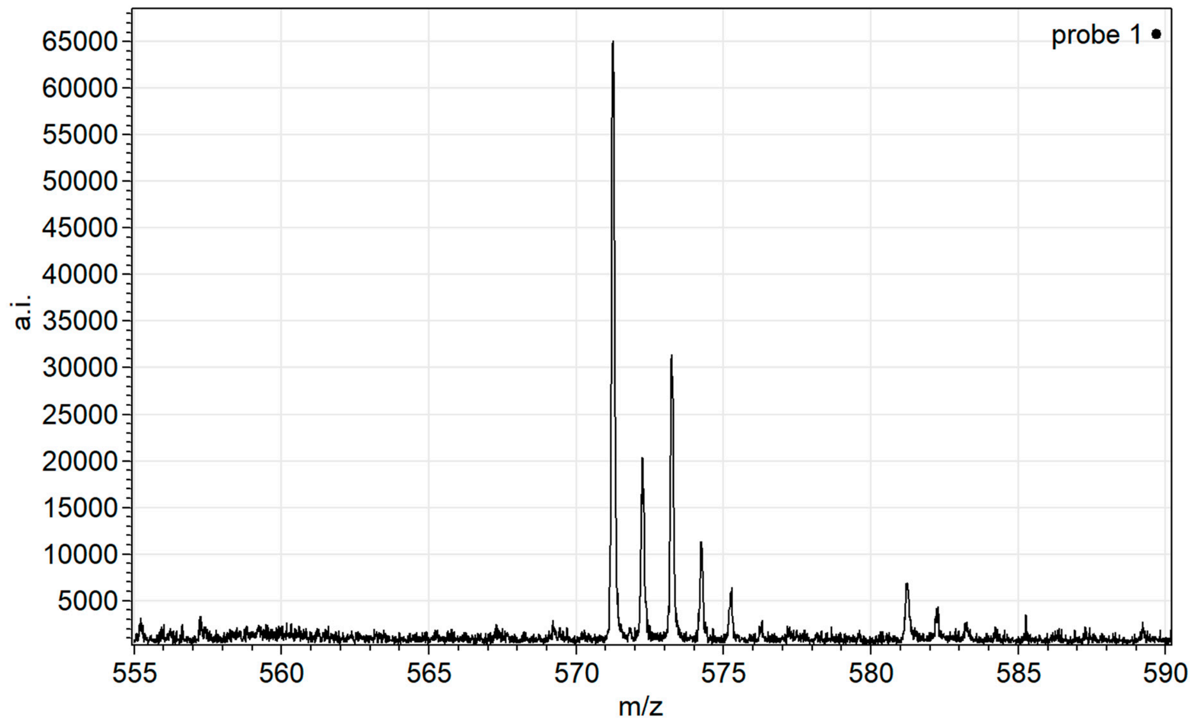

Figure S15 – MALDI-TOF spectrum of probe **1**

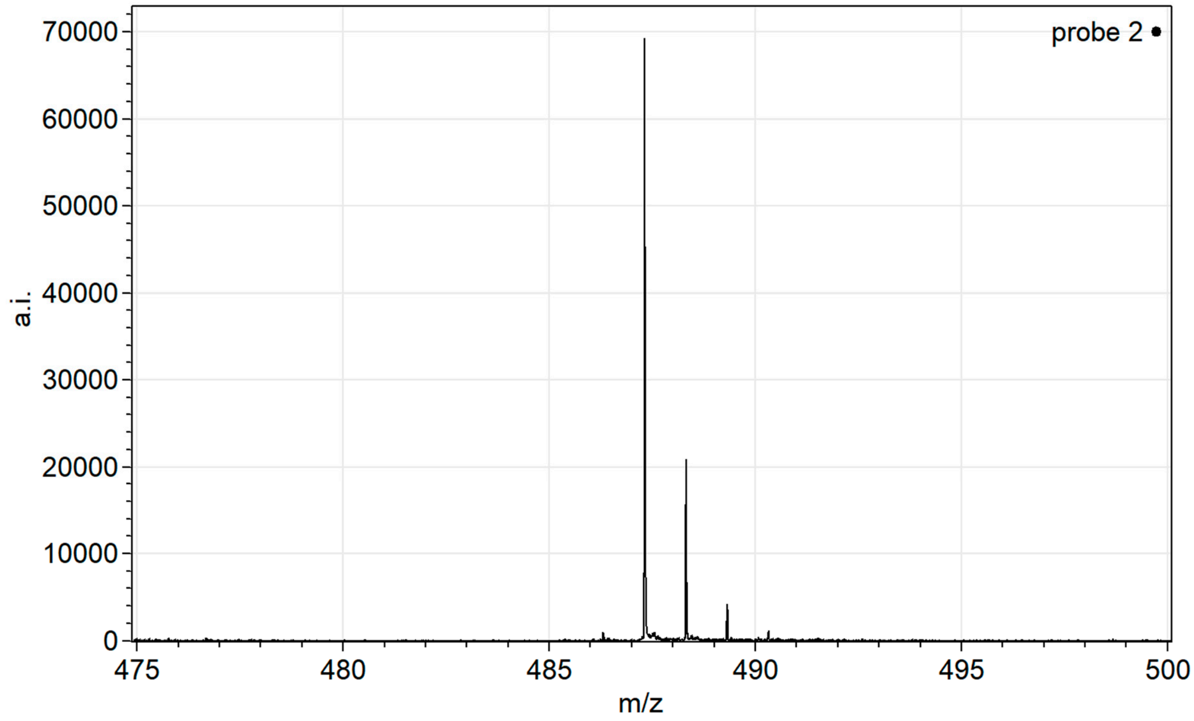

Figure S16 – MALDI-TOF spectrum of probe **2**

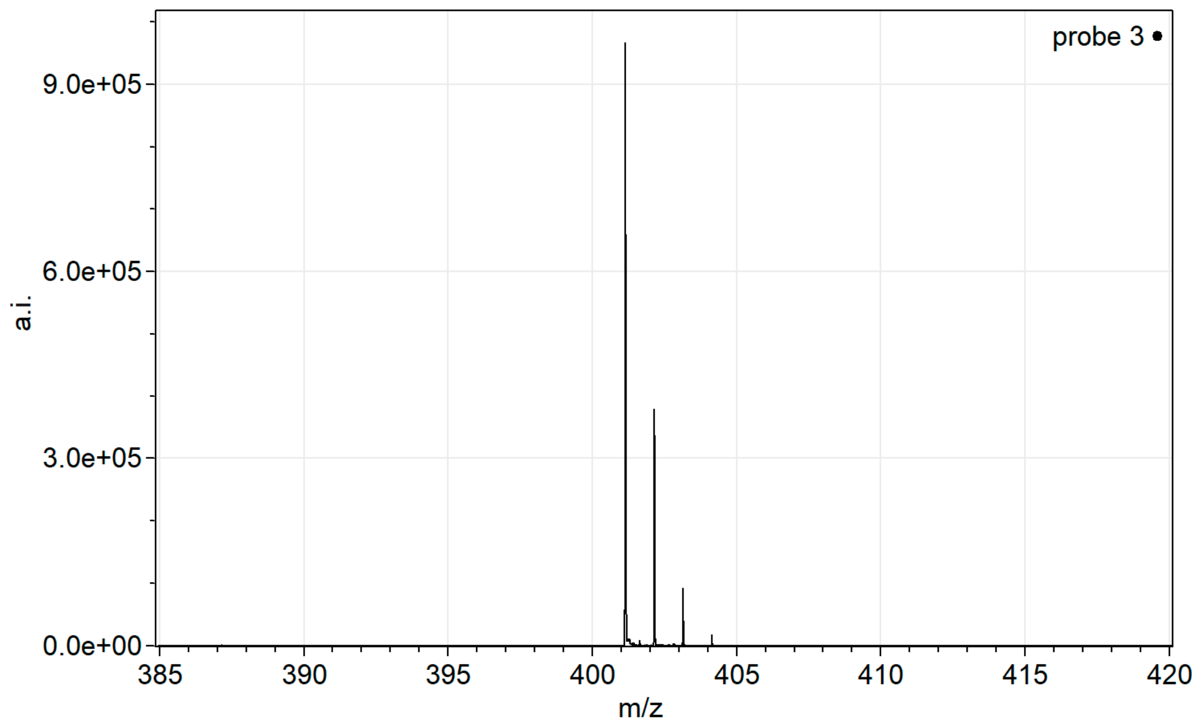

Figure S17 – MALDI-TOF spectrum of probe **3**
